# Putrescine Depletion Affects Arabidopsis Root Meristem Size by Modulating Auxin and Cytokinin Signaling and ROS Accumulation

**DOI:** 10.3390/ijms22084094

**Published:** 2021-04-15

**Authors:** Ahmed M. Hashem, Simon Moore, Shangjian Chen, Chenchen Hu, Qing Zhao, Ibrahim Eid Elesawi, Yanni Feng, Jennifer F. Topping, Junli Liu, Keith Lindsey, Chunli Chen

**Affiliations:** 1 College of Life Science and Technology, Huazhong Agricultural University, Wuhan 430070, China; ahmedhashem@webmail.hzau.edu.cn (A.M.H.); simon.moore@durham.ac.uk (S.M.); chenshangjian@webmail.hzau.edu.cn (S.C.); xccb456@webmail.hzau.edu.cn (C.H.); qing.zhao@webmail.hzau.edu.cn (Q.Z.); ibrahimeid@webmail.hzau.edu.cn (I.E.E.); feng@mail.hzau.edu.cn (Y.F.); 2 Key Laboratory of Plant Resource Conservation and Germplasm Innovation in Mountainous Region (Ministry of Education), Institute of Agro-Bioengineering, College of Life Science, Guizhou University, Guiyang 550025, China; 3 Biotechnology Department, Faculty of Agriculture, Al-Azhar University, Cairo 11651, Egypt; 4 Department of Biosciences, Durham University, South Road, Durham DH1 3LE, UK; j.f.topping@durham.ac.uk (J.F.T.); junli.liu@durham.ac.uk (J.L.); keith.lindsey@durham.ac.uk (K.L.); 5 Agricultural Biochemistry Department, Faculty of Agriculture, Zagazig University, Zagazig 44511, Egypt

**Keywords:** polyamine, root meristem, hormone signaling, ROS, auxin response, PIN transporter, cytokinin response

## Abstract

Polyamines (PAs) dramatically affect root architecture and development, mainly by unknown mechanisms; however, accumulating evidence points to hormone signaling and reactive oxygen species (ROS) as candidate mechanisms. To test this hypothesis, PA levels were modified by progressively reducing ADC1/2 activity and Put levels, and then changes in root meristematic zone (MZ) size, ROS, and auxin and cytokinin (CK) signaling were investigated. Decreasing putrescine resulted in an interesting inverted-U-trend in primary root growth and a similar trend in MZ size, and differential changes in putrescine (Put), spermidine (Spd), and combined spermine (Spm) plus thermospermine (Tspm) levels. At low Put concentrations, ROS accumulation increased coincidently with decreasing MZ size, and treatment with ROS scavenger KI partially rescued this phenotype. Analysis of double *AtrbohD/F* loss-of-function mutants indicated that NADPH oxidases were not involved in H_2_O_2_ accumulation and that elevated ROS levels were due to changes in PA back-conversion, terminal catabolism, PA ROS scavenging, or another pathway. Decreasing Put resulted in a non-linear trend in auxin signaling, whereas CK signaling decreased, re-balancing auxin and CK signaling. Different levels of Put modulated the expression of PIN1 and PIN2 auxin transporters, indicating changes to auxin distribution. These data strongly suggest that PAs modulate MZ size through both hormone signaling and ROS accumulation in *Arabidopsis*.

## 1. Introduction

Polyamines (PAs) are small polycationic compounds found in all living organisms. Due to their characteristic positive charges, they can interact with negatively charged molecules such as DNA, RNA, proteins, and phospholipids, and therefore influence their activity [1]. Putrescine (Put), spermidine (Spd), spermine (Spm), and thermospermine (Tspm) are the most common polyamines found in plants [2,3]. PAs have been shown to be implicated in the regulation of several plant physiological processes, including flower development, embryogenesis, organogenesis, senescence, and fruit maturation and development [4], and are also involved in *Arabidopsis* meristem development [5] and stress responses [6,7,8]. Recent studies on plant polyamines have been reviewed [9]. Put is the central diamine substrate compound for the biosynthesis of higher triamine and tetraamine polyamines. Unlike many other plants, *Arabidopsis thaliana* has only one Put biosynthesis pathway, through arginine decarboxylase [10]. In *Arabidopsis*, arginine decarboxylase 1 (ADC1) and ADC2 are key rate-limiting enzymes in Put synthesis, which catalyze L-arginine conversion into Put [10]. It was recently reported that the *ADC1* gene is involved in *N*-acetylputrescine biosynthesis from *N*δ-acetylornithine [11].

Cellular PA homeostasis is controlled by a combination of mechanisms, including transcriptional and upstream open reading frames (uORFs), translational regulation of PA biosynthesis genes, conjugation, back-conversion, and terminal catabolism [12,13,14]. The intricacies of PA biosynthesis are exhibited by several examples—treatment with D-Arg, the ADC-specific competitive inhibitor, results in a reduction in Put and increased Spd and Spm content in *Pringlea antiscorbutica* [15]; *adc2* mutants with low Put content show no change in Spd and Spm levels [16]; overexpression of *ADC* in plants generally results in high Put accumulation but in many cases exhibits relatively small changes in Spd and Spm [17]; and silencing of *ADC* genes significantly reduces Put, Spd, and Spm + Tspm in *Arabidopsis* ecotype Wassilewskija (Ws) [18]. This indicates that PA levels are under strict regulation [19] and emphasizes the complexity of the PA metabolic pathway.

Modifications in PA content can differentially affect root growth and development. For example, treatment with D-Arginine (D-Arg), a specific ADC1/2 competitive inhibitor, leads to a reduction in Put and a longer primary root length in *Pringlea antiscorbutica* [15]; perturbation of Put biosynthesis in *adc1* and *adc2* single T-DNA mutants shows no root phenotype, whereas the double mutant is lethal [20]; mutation in *BUD2* gene, which encodes S-adenosylmethionine decarboxylase 4 (SAMDC4), a key enzyme required for PA biosynthesis in *A. thaliana*, resulted in Put accumulation and altered root architecture [21]; silencing both *ADC1* and *ADC2* shows a significant reduction in primary root length [18], and high levels of Put treatment exhibit a similar phenotype in *Arabidopsis* [22]. Exogenous application of low levels of Put has no effect on root growth in *Arabidopsis* [23,24], but treatment with 1 mM Put increases root length in strawberries [25]. Treatment with an inhibitor of Tspm biosynthesis increased primary root growth, whereas treatment with exogenous Tspm inhibited root growth [26]; and inhibition or induction of *Arabidopsis* polyamine oxidase 5 (AtPAO5) significantly affected root length and development [27]. Such findings indicate complex regulatory effects of PAs on root development.

The PA back-conversion and terminal catabolism processes are mediated by two classes of amine oxidases, copper-containing amine oxidases (CuAOs) and FAD-dependent polyamine oxidases (PAOs). These enzymatic reactions lead to the production of H_2_O_2_ [8,28,29,30], suggesting another mechanism by which PAs can affect root growth and development [28], in addition to their role as ROS scavengers [31], acting to reduce stress-induced ROS accumulation in leaves and roots [32,33].

ROS homeostasis plays a vital role in root growth and development. A study of how changes in ROS accumulation affect root development [34] reported that the balance between hydrogen peroxide (H_2_O_2_) and superoxide (O_2_^−^) affects root growth and meristem structure by influencing the transition from cell division to cell differentiation. It was also demonstrated that perturbation of polyamine catabolism ZmPAO1 strongly influences root development and xylem differentiation in *Zea mays*, mediated by H_2_O_2_ production [35]. Such studies suggest that altering ROS homeostasis is one mechanism by which PAs could affect root development.

Several studies indicate that PAs also modify hormone response and crosstalk under specific physiological and developmental processes [36,37]; for example, transcriptome studies revealed that changes in endogenous PA content altered the expression levels of genes associated with biosynthesis and signaling of several plant hormones such as auxin, ethylene, and gibberellins [38]; it is necessary for interactions between Tspm, auxin, and cytokinin to be tightly controlled for proper xylem development and plant growth [27]; PAs and auxin affect root growth and development in two sweet orange cultivars [39]; and several studies have further explored the relationships between PAs and auxin response [40]. The above data provide evidence to suggest that the adjustment of the hormone response is an additional mechanism by which PAs affect root development.

The above studies demonstrate that changes in PA levels can influence plant phenotypes in multiple species and tissues; however, mechanisms linking PA levels to phenotype are not well understood. In this work, we focus on the root meristem to identify PA-driven mechanisms that regulate the meristem phenotype under conditions of Put depletion in *Arabidopsis*.

## 2. Results

**Figure 1:**
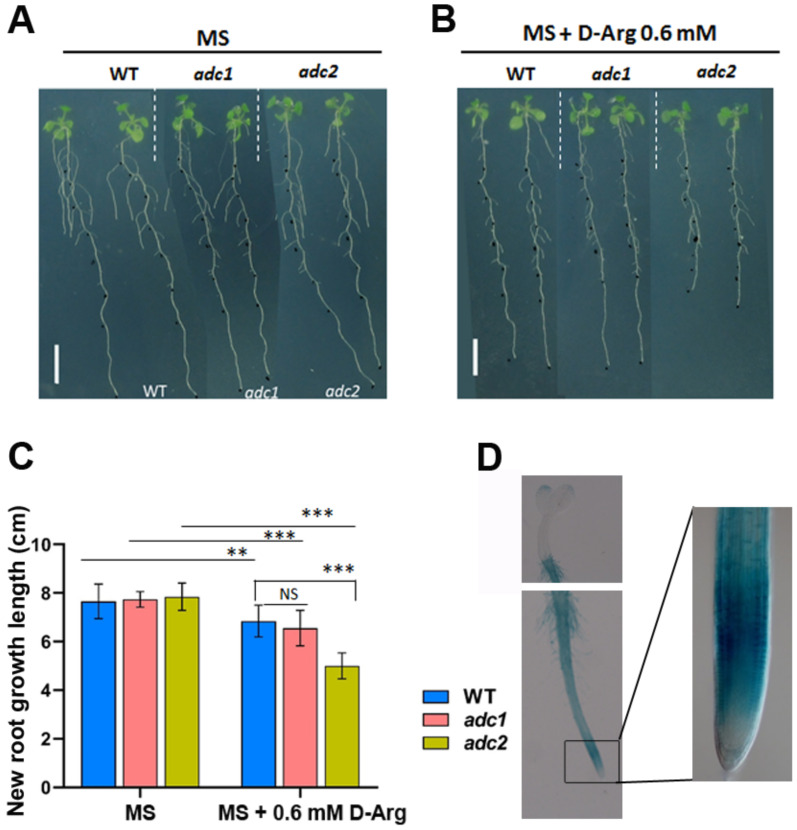
*adc1* and *adc2* single mutants exhibit no root phenotype and the results indicate that ADC2 localizes mainly to the root and *adc2* is more sensitive to D-Arg than *adc1*. For (**A**), (**B**) and (**C**), 5-day-old seedlings at the same developmental stage were transferred to new media containing water (Ctrl) or 0.6 mM D-Arg and were treated for 8 days. (**A**) Untreated wild-type (WT), *adc1,* and *adc2* root phenotypes are the same. Scale bar = 1 cm. (**B**) D-Arg treatment inhibits root length of WT, *adc1*, and *adc2*. Scale bar = 1 cm. (**C**) D-Arg treatment exhibits no difference between WT and *adc1* root length; however, the *adc2* root is significantly shorter that of WT and *adc1* phenotypes. (**D**) Histochemical localization of GUS activity in 2-day-old transgenic seedling harboring promoter ADC2::GUS (*pAtADC2::GUS*). Data shown are averages ± SD (n > 30). Asterisks denote significant differences (** *p* < 0.01, *** *p* < 0.001; Student’s *t*-test).

Since *adc1* and *adc2* single T-DNA mutants exhibit no root phenotype (Figure 1A,C) and the double mutant is lethal, it was decided to adjust PA levels by simultaneously reducing the activity of ADC1 and ADC2, which are key enzymes in the Put biosynthesis pathway. Several previous studies [41,42,43,44,45,46] have used D-Arg treatment to inhibit ADC1/2 and deplete Put levels. We progressively reduced Put biosynthesis, the substrate of Spd, Spm, and Tspm, by inhibiting ADC1/2 enzyme activity using the competitive inhibitor D-Arg, which specifically inhibits ADC1/2 activity by blocking binding of the Put substrate L-Arg. Given the specificity of D-Arg and its mode of action, this method was thought to be equivalent to using ADC genetic knockdowns, with the additional advantage of allowing improved flexibility in the control over ADC activity and Put biosynthesis.

*ADC2::GUS* results confirmed the differential localization of ADC2 expression to the root (Figure 1D). The results also suggested that ADC2 activity is higher in the root than that of ADC1, since while D-Arg treatment inhibited root length in WT, *adc1*, and *adc2*, D-Arg inhibition of ADC1 activity in the *adc2* mutant resulted in a shorter root than inhibition of ADC2 did in the *adc1* mutant, indicating that the *adc2* mutant root phenotype is more sensitive to D-Arg than *adc1* (Figure 1B,C).

### 2.1. D-Arg Application Promotes or Inhibits Root Growth and Meristem Size in a Concentration-Dependent Manner

**Figure 2:**
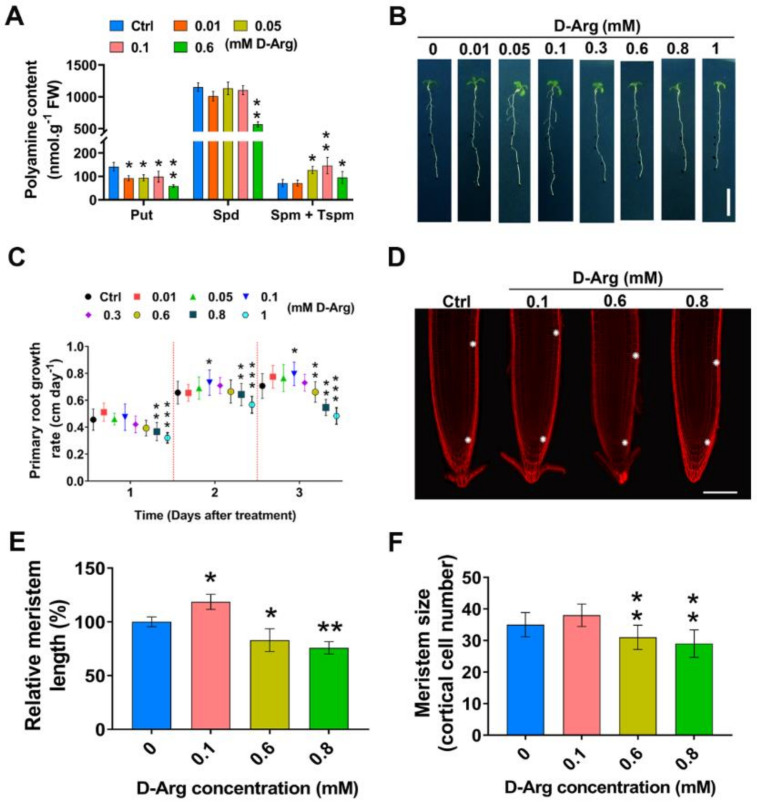
ADC1/2 inhibition differentially modifies polyamine (PA) content and affects root growth and meristematic zone (MZ) size in a non-linear concentration-dependent manner. Five-day-old seedlings at the same developmental stage were transferred to new media containing water (Ctrl) or D-Arg and were treated for 3 days. (**A**) Differential effect of D-Arg treatment on polyamine content. Putrescine (Put), spermidine (Spd), and spermine plus thermo-spermine (Spm + Tspm) levels were measured using HPLC. (**B**) Whole seedling phenotype shows a non-linear effect of increased D-Arg treatment. Scale bar = 1 cm. (**C**) Daily new root growth length varies for different D-Arg concentrations. (**D**) Root meristem imaging, using PI staining and confocal microscopy, exhibits the non-linear effects of D-Arg on MZ size. Two white asterisks on each root indicate the quiescent center (QC) (bottom) and first elongated cell (top). Scale bar = 100 μm. (**E**) Meristem cell length normalized to control. (**F**) Cortical cell number in the meristem zone. Data shown are averages ± SD (n > 30). Asterisks denote significant differences compared with the control (* *p* < 0.05, ** *p* < 0.01; Student’s *t*-test).

To explore how Put depletion modulates root growth and development, we treated 5-day-old *Arabidopsis* wild-type Col-0 seedlings for a further 3 days with a range of concentrations from 0.01 mM to 1 mM D-Arg, a competitive inhibitor of ADC1/2 activity (Figure 2A,B). To determine the effect of D-Arg on polyamine content, seedlings were treated with 0.01, 0.05, 0.1, and 0.6 mM of D-Arg for three days. Whole seedlings were used for HPLC analysis. Data revealed a significant reduction in Put of approximately 30–35% upon 0.01, 0.05, and 0.1 mM treatment, and then a significant decrease of approximately 60% upon 0.6 mM treatment. The Spm plus Tspm concentration was unchanged with 0.01 mM D-Arg treatment, and it increased by 78% at 0.05 mM, by 100% at 0.1 mM, and then only by 35% at 0.6 mM treatment, relative to controls (Figure 2A). Interestingly, Spd remained stable until a considerable decrease in Put occurred at 0.6 mM D-Arg, when Spd declined by approximately 50% (Figure 2A). Spd levels appeared to be more tightly controlled than for Put and Spm + Tspm.

After 3 days of treatment, we observed an inverted-U trend in root growth as D-Arg concentrations increased (Figure 2B,C). Compared to controls, root growth was unchanged at 0.01 mM and 0.05 mM treatments, enhanced at 0.1 mM, unchanged at 0.3 mM, and then root growth gradually decreased at treatment concentrations of 0.6 mM and greater.

During the 3-day D-Arg treatment period, we observed interesting changes in daily root growth as demonstrated by the new root growth-rate curve (Figure 2C). We noticed no significant change in root length at concentrations from 0.01 to 0.6 after one day of treatment, whereas 0.8 and 1 mM showed a significant decrease. However, the root growth rate increased at days two and three at 0.1 mM and then started to decline at 0.8 and 1 mM. The root reduction on day three became highly significant, at 0.6 mM and more, compared to untreated controls.

Root growth is the result of both cell division in the meristem zone (MZ) and cell expansion in the elongation zone [47]. We chose to more closely examine the meristem structure under D-Arg treatments with a series concentration (Figure 2D) and measured the meristem length and number of cells in the MZ cortical layer. Root cells were analyzed by means of differential interference contrast (DIC) microscopy and compared to the untreated control. A medium D-Arg concentration of 0.1 mM enlarged the meristem by approximately 18%, and MZ size decreased by 20% at 0.6 mM D-Arg and by 25% at 0.8 mM D-Arg (Figure 2E). The MZ cell number slightly increased, but not significantly, at 0.1 mM and then decreased significantly at 0.6 and 0.8 mM D-Arg (Figure 2F).

### 2.2. KI Application Indicates That MZ Size Inhibition at High D-Arg Concentration Is Partially Due to H_2_O_2_ Accumulation

**Figure 3:**
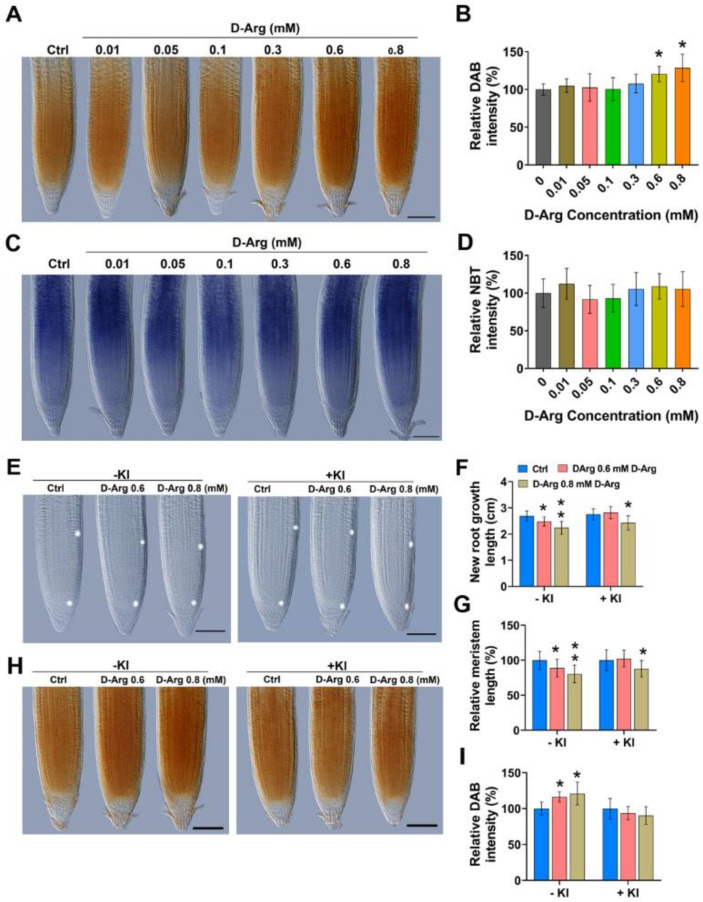
D-Arg-induced H_2_O_2_ accumulation, root length, and MZ size reduction is partially rescued by ROS scavenging with 1 mM KI. Five-day-old seedlings at the same developmental stage were transferred to new media containing water (Ctrl) or D-Arg and treated for 3 days. MZ imaging by differential interference contrast (DIC) microscopy. (**A**,**B**) 3,3′-diaminobenzidine (DAB) staining and relative stain intensity indicate that D-Arg treatment increases H_2_O_2_ accumulation in root meristem. (**C**,**D**) Nitrotetrazolium blue (NBT) staining and relative stain intensity indicates that D-Arg treatment does not change O_2_^−^ accumulation in the root meristem. (**E**–**G**) Treatment with and without D-Arg and 1 mM KI shows partial rescue of root meristem and root length by KI. (**H**,**I**) DAB staining of root meristem and relative stain intensity, with or without D-Arg and KI, show successful H_2_O_2_ scavenging by KI. All scale bars = 100 µm. Data shown are averages ± SD (n > 30). Asterisks indicate significant differences compared with the control (* *p* < 0.05, ** *p* < 0.01; Student’s *t*-test).

To investigate whether treatment with different D-Arg concentrations alters ROS accumulation in WT seedlings, we used 3,3′-diaminobenzidine (DAB) (Figure 3A) and nitrotetrazolium blue (NBT) (Figure 3C) staining to detect the presence of H_2_O_2_ and O_2_^−^ respectively in vivo. DAB staining indicated no difference in ROS accumulations at low and medium D-Arg treatment, but at high D-Arg treatment of 0.6 mM and 0.8 mM, ROS increased significantly (Figure 3B). Furthermore, we did not detect significant changes in NBT staining at any D-Arg concentration (Figure 3D).

To determine whether the reduction in root length and root meristem at higher D-Arg concentrations was attributable to H_2_O_2_ accumulation, we exposed control roots and roots treated with 0.6 mM and 0.8 mM D-Arg to the ROS scavenger potassium iodide (KI, 1 mM), as described previously [34]. KI application successfully scavenged H_2_O_2_ (Figure 3H,I) and completely rescued the shorter root phenotype caused by 0.6 mM of D-Arg treatment, whereas at 0.8 mM D-Arg, root length and meristem size were only partially recovered (Figure 3E–G), suggesting that at higher D-Arg levels, reduced root length is not completely ROS-dependent and that other factors are involved.

### 2.3. H_2_O_2_ Accumulation Is Not Caused by NADPH Oxidases

**Figure 4:**
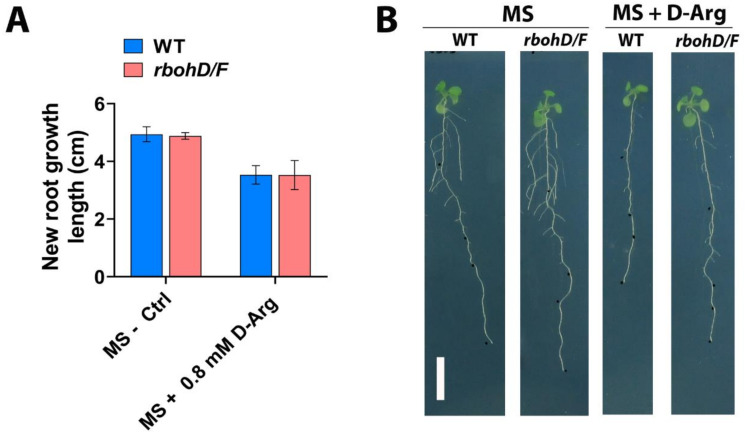
Effect of D-Arg treatment on WT and *AtrbohD/F* root growth indicates that H_2_O_2_ accumulation is not caused by NADPH oxidases. Five-day-old seedlings at the same developmental stage were transferred to new media containing water (Ctrl) or 0.8 mM D-Arg and treated for 6 days. (**A**) New root growth length of WT and *AtrbohD/F*. (**B**) Seedling phenotype. Scale bar = 1 cm. Data shown are averages ± SD (n > 15).

During plant growth and development, ROS are predominantly generated by the NADPH oxidases RBOHD and RBOHF class III peroxidases [48,49] and polyamine amine oxidases (PAO) [8]. Some reports show a link between PAs and NADPH oxidases (RBOHD/F) in tobacco and *Arabidopsis* [50,51]. To investigate the involvement of NADPH oxidases in H_2_O_2_ accumulation at high D-Arg treatments, we examined the phenotype of seedlings carrying double *atrbohD/F* loss-of-function mutants. In this set of experiments, the D-Arg treatment period was extended from 3 to 6 days and new root growth length was observed to ensure that NADPH oxidases were not a significant source of ROS accumulation under conditions of Put depletion. The results showed that, similarly to the WT results, exogenous high D-Arg application inhibited the new root growth of *atrbohD/F* (Figure 4A,B).

### 2.4. Auxin Response Exhibits a Non-Linear Trend as ADC1/2 Activity Decreases

**Figure 5:**
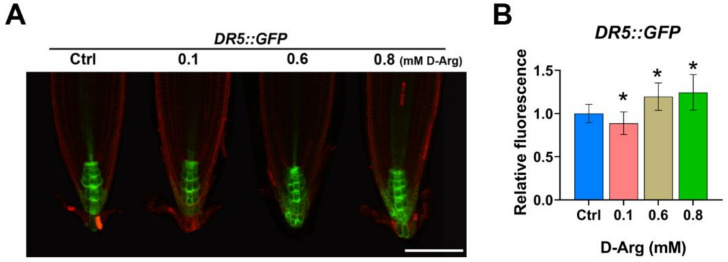
D-Arg treatment has a non-linear effect on auxin response. Five-day-old seedlings at the same developmental stage were transferred to new media containing water (Ctrl) or D-Arg and treated for 3 days. (**A**) Confocal images of *DR5::GFP* auxin response reporter. (**B**) *DR5::GFP* normalized fluorescence indicates a non-linear auxin response trend. Data are averages ± SD (n > 15). Scale bar = 100 μm. Asterisks denote significant differences compared with the control (* *p* < 0.05; Student’s *t*-test).

Auxin signaling promotes cell division and is involved in regulating root meristem size [47]. We analyzed the effects of Put depletion on the auxin response using the reporter line *DR5::GFP.* Interestingly, increasing levels of exogenous D-Arg had a non-linear effect on *DR5*-dependent GFP fluorescence (Figure 5A). We observed that 0.1 mM D-Arg decreased *DR5* activity by 15%, but in contrast, higher D-Arg at 0.6 and 0.8 mM significantly enhanced it by 20–25%, respectively (Figure 5B).

### 2.5. CK Response Gradually Decreases as D-Arg Increases

**Figure 6:**
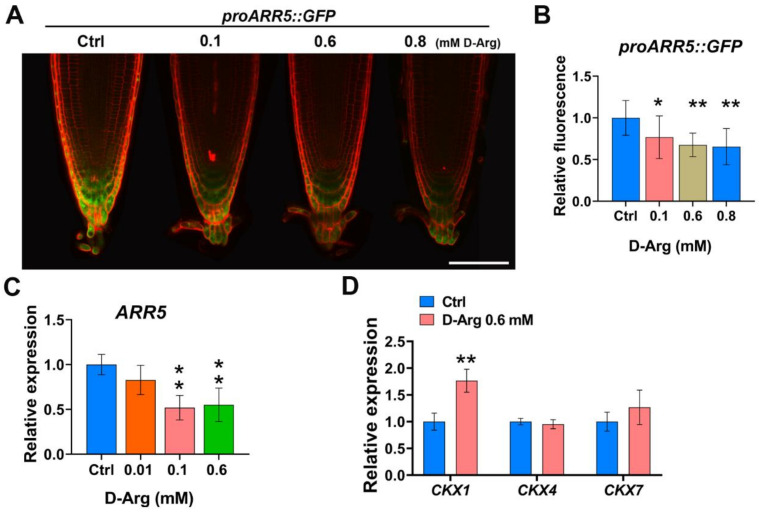
D-Arg treatment reduces the cytokinin response and increases *CKX1* expression in the root meristem. Five-day-old seedlings at the same developmental stage were transferred to new media containing water (Ctrl) or D-Arg and treated for 3 days. (**A**,**B**) Confocal imaging indicates that increasing D-Arg gradually reduces cytokinin response *proARR5::GFP* and relative *proARR5::GFP* fluorescence. Scale bar = 100 μm. (**C**,**D**) qPCR data indicate that D-Arg treatment modulates the relative expression of *ARR5* and cytokinin oxidase (*CKX*) genes. Data shown are averages ± SD (n > 15). Asterisks indicate significant differences compared with the control (* *p* < 0.05, ** *p* < 0.01; Student’s *t*-test).

Cytokinin (CK) signaling promotes cell differentiation and negatively regulates root meristem size [47]. To investigate whether reduced Put affects CK signaling, we used the reporter line *proARR5::GFP*. Arabidopsis response regulator 5 (ARR5) is a type-A negative regulator of cytokinin response and is strongly induced by CK [52]. Under different levels of D-Arg treatment, we observed that *proARR5::GFP* fluorescence gradually decreased (Figure 6A). The CK response was reduced significantly at 0.1 mM D-Arg by 25% and at higher D-Arg levels of 0.6 mM and 0.8 mM by 33% to 35% compared to the untreated control (Figure 6B). *ARR5* expression was measured using qRT-PCR at different concentrations of applied D-Arg. At low (0.01 mM) D-Arg, we observed a slight but not significant reduction in the ARR5 transcript level (Figure 6C). Similarly to the *proARR5::GFP* results, medium and high levels of D-Arg, at 0.1 mM and 0.6 mM, significantly downregulated *ARR5* transcription by approximately 50%, indicating that D-Arg treatment and Put depletion progressively reduces the CK response in the *Arabidopsis* root. Cytokinin activity is regulated by the balance between biosynthesis and degradation [53,54]. Cytokinin oxidase/dehydrogenases (CKXs) play a key role in CK degradation [54]. It has been reported that CKX family genes play critical roles in determining root architecture in several plant species [55,56,57,58,59]. In *Arabidopsis*, AtCKX1, AtCKX4, and AtCKX7 have been shown to either localize to the root or their overexpression has been shown to affect root phenotype [53,56,60,61]; however, this does not preclude other members of the CKX family playing a significant role in root development. Our results reveal that ADC1/2 inhibition significantly induced cytokinin oxidase1 (*CKX1*) expression levels and slightly induced *CKX7* in the root, whereas *CKX4* was unchanged (Figure 6D).

### 2.6. Differential Modulation of PIN1 and PIN2 Protein at Low and High D-Arg Treatment

**Figure 7:**
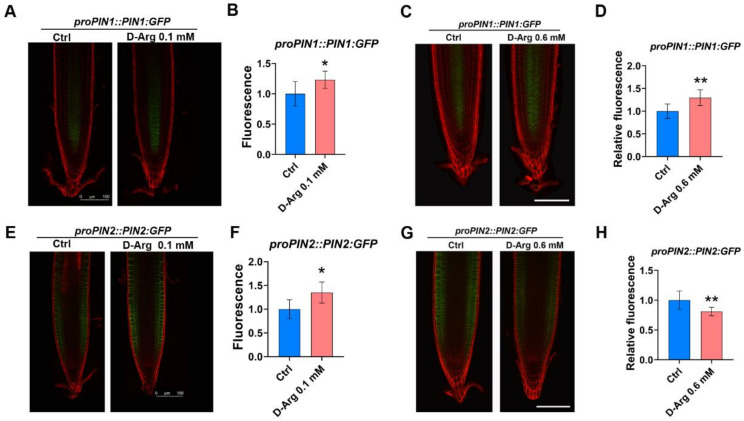
D-Arg treatment differentially affects PIN1 and PIN2 auxin efflux carriers. Five-day-old seedlings at the same developmental stage were transferred to new media containing water (Ctrl) or 0.6 mM D-Arg and treated for 3 days. (**A**,**B**) Confocal imaging of *proPIN1::PIN1:GFP* reporter line and relative *proPIN1::PIN1:GFP* fluorescence indicate that low D-Arg treatment increases PIN1 protein. (**C**,**D**) *proPIN1::PIN1:GFP* reporter line and relative *proPIN1::PIN1:GFP* fluorescence indicate that high D-Arg treatment increases PIN1. (**E**,**F**) *proPIN2::PIN2:GFP* reporter line and relative *proPIN2::PIN2:GFP* fluorescence indicate that low D-Arg treatment increases PIN2 protein. (**G**,**H**) *proPIN2::PIN2:GFP* reporter line and relative *proPIN2::PIN2:GFP* fluorescence indicate that high D-Arg treatment reduces PIN2 protein. All scale bars =100 µm. Data shown are averages ± SD (n > 15). Asterisks indicate significant differences compared with the control (* *p* < 0.05, ** *p* < 0.01; Student’s *t*-test).

We explored the level of protein and localization of auxin efflux transporters PIN1 and PIN2 using two reporter lines, *proPIN1::PIN1:GFP* and *proPIN2::PIN2:GFP*. The auxin efflux transporter PIN1 is localized to the vascular tissue membrane of the root meristem and is involved in auxin transport from the stele to the quiescent center and columella initials [62]; PIN2 is localized mainly in the outer cell layers and is an essential component for basipetal auxin transport [63], and both PIN1 and PIN2 are important for correct auxin distribution [63]. Confocal imaging at low levels of D-Arg revealed significant increases in *PIN1:GFP* by 23% (Figure 7A,B) and *PIN2:GFP* by 35% (Figure 7E,F) compared to untreated controls. At high D-Arg, the results showed that the level of *PIN1:GFP* was significantly increased by 30% compared to the untreated control (Figure 7C,D). In contrast, *PIN2:GFP* at the same D-Arg concentration was significantly decreased by 20% (Figure 7G,H). Our results therefore suggest that Put depletion alters PIN1 and PIN2 levels, cellular auxin efflux, and auxin distribution.

## 3. Discussion

The goal of our study was to investigate the effect of PAs on root growth and development in *Arabidopsis* and identify possible mechanisms by which PAs affect root phenotype under conditions of ADC1/2 inhibition. Under normal conditions, *ADC1* expression is localized to the shoot and *ADC2* expression to the root [64]. Our *ADC2::GUS* results (Figure 1D) confirm that *ADC2* expression is localized to the root. Given that neither *adc1* or *adc2* single mutants exhibited a root phenotype (Figure 1A), it is reasonable to assume that Put levels, directly or indirectly, affect the localization and/or level of *ADC1/2* expression. This assumption is supported by the literature, which provides evidence of *ADC1/2* regulation by both PAs and hormones [37,65,66,67,68,69]. To identify links between Put depletion and MZ size, we therefore used D-Arg application to simultaneously inhibit both ADC1 and ADC2 to ensure a reduction in Put levels, rather than developing specific *ADC2* knockdowns. Although *ADC1/2* regulation, localization, or redundancy are important components of this complex biological system, it was not part of this initial study but could be addressed in future research.

PA levels were modified by using the specific ADC1/2 enzyme inhibitor D-Arg to perturb PA biosynthesis instead of using PA treatment, since exogenous application, for example of Spd, could artificially increase Spd levels, affect PA balance, and generate ROS due to reverse biosynthesis and terminal catabolism [8,28,29]. D-Arg application has been used to deplete Put in several research papers [41,42,43,44,45,46]; however, none of these papers addressed the issue of whether D-Arg application could produce any significant additional effects by, for instance, regulation of other enzyme activities or by increasing the availability of the ADC1/2 substrate, L-Arg. This is an outstanding issue for future research, which is discussed further in the conclusion.

Inhibition of ADC1/2 reduced Put levels and resulted in unpredictable changes in the levels of higher PAs. As Put levels decreased, Spd remained stable until high D-Arg application, when levels dropped significantly, and Spm + Tspm levels initially increased and then decreased at high D-Arg. These unexpected and differing trends in PA levels under conditions of progressive ADC1/2 inhibition demonstrate the complexity of the PA forward and reverse biosynthesis pathways that re-balance PA levels when Put is depleted. A more detailed examination of how PA levels change under different conditions of enzyme inhibition, PA treatment, or under stress conditions could prove useful in investigating the regulation of PA balancing.

Put depletion resulted in non-intuitive phenotype outcomes, with increased MZ size observed at low levels of D-Arg application and progressively decreasing MZ size at higher D-Arg. This inverted-U trend in MZ size suggests that more than one mechanism regulates MZ size: for example, with one mechanism promoting MZ size at low D-Arg concentrations and another antagonistic mechanism inhibiting MZ size at higher D-Arg levels. At low D-Arg levels the first mechanism acts to increase MZ size, but as D-Arg treatment increases, the second mechanism becomes dominant in order to reduce MZ size.

It has been shown that root MZ size is determined by the balance between cell division and differentiation and that this process is regulated by two mechanisms—auxin and CK signaling balance [47], and changes in ROS homeostasis [34] that are independent of auxin and CK signaling. We therefore examined these two candidate mechanisms. Since auxin promotes cell division, whereas CK promotes differentiation, the auxin-to-CK signaling balance modulates MZ size [47], rather than individual auxin or CK signaling trends. Auxin signaling displayed a U-shaped trend as D-Arg increased, whereas cytokinin progressively decreased. At low D-Arg application, no change in ROS accumulation was observed, but the auxin:CK ratio shifted in favor of auxin to enlarge the MZ, consistent with the literature [47]. However, at higher D-Arg levels, we observed increasing ROS accumulation, which is known to inhibit MZ size [34,70,71,72]. The results for DAB and NBT staining (Figure 3D) suggest that high Put depletion led to the accumulation of H_2_O_2_ but not of O_2_^−^ and that that H_2_O_2_ accumulation is caused by PAO activity, rather than by NADPH oxidases (Figure 4). The ROS effect was confirmed with phenotype rescue by ROS scavenging using KI; however, the partial rescue at high D-Arg also suggests that additional mechanism(s) could be involved in MZ size and root growth regulation.

We concluded that the hormone effect promoted MZ size at low D-Arg but that at higher D-Arg the antagonistic ROS effect dominated to reduce the MZ. The underlying mechanisms whereby Put depletion modulates ROS accumulation and auxin and cytokinin signaling have yet to be unraveled; however, the literature points to several promising lines of investigation: (1) previous studies report that PAs play two opposing roles in the regulation of ROS levels as both ROS scavengers under stress [32,33] and as a source of ROS generated by terminal and back conversion pathways [8,28,29], (2) Spd and Spm have been shown to regulate CKX [37,38,73] and auxin conjugation [37], and (3) Tspm inhibits the final steps in the CK biosynthesis pathway [3,74].

The results of our work indicate that Put depletion affects root phenotype through the antagonistic actions of hormone signaling and ROS accumulation; however, the question remains as to whether this is due to direct or indirect effects of changing Put levels. As noted earlier, PA levels are closely linked by forward and reverse biosynthesis pathways. Furthermore, terminal catabolism of excess PAs and back-conversion to lower-level PAs can produce bioactive products. These mechanisms constitute an extremely complex process by which perturbation of a single PA can result in changes in other PAs and in the generation of bioactive products. This is in part illustrated by our HPLC experiments, which produced non-intuitive results in regard to the changes in higher PAs when Put was depleted. It is therefore likely that perturbation of any single PA results in PA re-balancing and effects phenotype outcomes by multiple direct and indirect pathways. Several studies also indicate that the overall PA balance seems to be more important than the specific effect of individual PAs [15,75].

Previous work [47] showed that changes in relative auxin to CK signaling modulate MZ size through the regulation of auxin transporters and modified auxin distribution. In our work, at low D-Arg the *proPIN1::PIN1:GFP* protein reporter showed an increase in PIN1 and the *proPIN2::PIN2:GFP* showed an increase in PIN2, whereas upon high D-Arg treatment, PIN1 increased and PIN2 decreased (Figure 7), indicating that ADC1/2 inhibition differentially modulates PIN1 and PIN2 protein levels to modify auxin transport and distribution.

**Figure 8:**
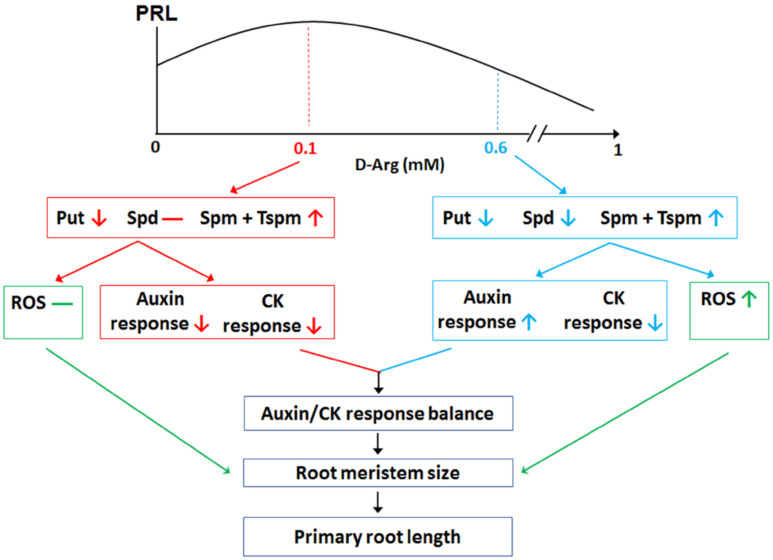
ADC1/2 inhibition differentially changes PA levels and has a non-linear effect on meristem size by modulating relative auxin/CK signaling and ROS accumulation. Exogenous application of increasing D-Arg concentrations differentially modified Put, Spd, and Spm + Tspm levels and resulted in an inverted-U trend in primary root growth. Medium D-Arg at 0.1 mM (red boxes) promoted root growth, decreased the auxin and CK response, and had no effect on ROS levels (green boxes). High D-Arg 0.6 mM (blue boxes) reduced root growth, differentially affected auxin and CK response, and accumulated ROS. PRL, primary root length. Vertical arrows denote a statistically significant increase↑ or decrease↓, whereas a horizontal line indicates no significant change.

The above data demonstrate that hormone signaling and ROS accumulation are two mechanisms by which PAs regulate MZ size (Figure 8).

## 4. Materials and Methods

### 4.1. Plant Materials and Growth Conditions

All experiments were performed using *Arabidopsis thaliana* ecotype Columbia (Col-0). Transgenic marker lines are in Col-0 backgrounds as follows: *DR5::GFP* for auxin-response; *proPIN1::PIN1:GFP* and *proPIN2::PIN2:GFP* for PIN1/2 auxin efflux carrier proteins; *proARR5::GFP* for cytokinin response; and *proADC2::GUS* for localization of *ADC2* expression. Genetic materials, T-DNA insertion mutant *adc1* and *adc2* for polyamine biosynthesis pathway and *atrbohD/F* for ROS experiments were used.

Seeds were surfaced-sterilized for 2 min in 75% ethanol and then 10 min in 1% sodium hypochlorite. Seeds were washed five times with sterilized deionized water. For simultaneous germination, all seeds were stratified for 2–3 days at 4 °C before germination.

Seeds were grown on 120 × 120 × 17 mm square plates containing half-strength Murashige and Skoog (MS) medium 2.2 g L^−1^ (Duchefa Biochemie) including vitamins, MES 0.5 g L^−1^ (Duchefa Biochemie), and 1% (*w/v*) sucrose and solidified with 1% agar (Duchefa Biochemie). PH was adjusted to 5.8 with KOH. Plated were sealed with Micropore tape.

To maintain the root for molecular assay and physical root measurements, seedlings were grown and placed vertically in the growth incubator at 22 °C with light cycles of 16 h light/8 h dark (light intensity: ∼120 μmol/s/m^2^, and relative humidity of 60%). For putrescine depletion, 5-day-old seedlings were transferred to MS medium containing 0, 0.01, 0.05, 0.1, 0.3, 0.6, 0.8, and 1 mM of D-Arginine (Sangon Biotech, Shanghai, China) for a further 3 days. For experiments involving double *atrbohD/F* loss-of-function mutants, the D-Arg treatment period was extended from 3 to 6 days and new root growth length was observed to ensure that NADPH oxidases were not a significant source of ROS accumulation under conditions of Put depletion.

### 4.2. Root Length, Root Meristem Size, and Cell Length Measurements

To track changes in root growth, five-day-old seedlings with the same root length were transferred to a fresh medium containing either water as control or D-Arginine. The position of the root tip was marked on the back of plates at the time of transfer and new root growth length was measured as root growth extension during the treatment period, using images taken with a Nikon D300s digital camera (Nikon Corp., Tokyo, Japan).

For root meristem measurements, after 3 days D-Arg treatment seedlings were cleared with chloral hydrate solution [76]. Root meristem size was determined as the length from the quiescent center (QC) to where the cell was double the size of the previous cell along the cortex cell layer [77,78]. Root meristems were analyzed and imaged using an Olympus BX61 microscope (Olympus, Tokyo, Japan) equipped with differential interference contrast (DIC) optics, 20x UPlanSApo objective, and a CCD camera (Olympus DP74). For Photo acquisition, Olympus cellSens software was used.

### 4.3. Confocal Laser Scanning Microscopy

All confocal root images were taken with a Leica SP8 confocal laser scanning microscope (https://www.leica-microsystems.com) after three days of D-Arg treatment. Ten micrograms per milliliter of propidium iodide (PI) solution (Sangon Biotech, Shanghai, China) were used to visualize cell walls. Stained roots were visualized with the excitation wavelength set at 548 nm for PI and at 488 nm for green fluorescent protein (GFP).

### 4.4. RNA Extraction, cDNA Synthesis, and qRT-PCR

Whole roots of Col-0 wild type non-treated (Ctrl) and treated with D-Arginine (D-Arg) were used. Total RNA extraction was performed using TransZol Up reagent (TransGen, Beijing, China). RNA concentration and quality were determined with a Nanodrop 2000 Spectrophotometer (Thermo Fisher Scientific). cDNA was synthesized from 1 µg of RNA using a TransScript One-Step gDNA Removal and cDNA Synthesis SuperMix kit (TransGen, Beijing, China), following the manufacturer’s instructions. cDNA was diluted 1:10 for quantitative real-time PCR (qPCR).

Quantitative real-time PCR analyses were performed using TransStart Tip Green qPCR SuperMix (TransGen, Beijing, China) with a Roche LightCycler 480 thermal cycler instrument, 384-well (Roche). Relative expression values were calculated using the 2^-ΔΔCt^ method [79], and ACTIN2 was used as a reference gene. Primers are listed in Table S1. Three biological replicates were performed for each sample and each biological replicate was represented by three technical replicates.

### 4.5. Quantification of Free Polyamine

Free PA quantification was performed using high-performance liquid chromatography (HPLC). Five-day-old seedlings at the same developmental stage were selected and transferred to the treatment media containing D-Arg for another 3 days. Zero point three five grams of fresh weight of seedlings were harvested for analysis. The extraction and quantification methods were performed as described in [80]. 1,6-hexanediamine was used as an internal standard.

### 4.6. Histochemical GUS Staining

Transgenic Arabidopsis containing ADC2 promoter::GUS fusions were stained for GUS according to the method described in [32].

### 4.7. Determination of O_2_^−^ and H_2_O_2_ by NBT and DAB Staining

3,3′-Diaminobenzidine (DAB, 1 mg mL^−1^) staining (Sigma Aldrich) was used to detect H_2_O_2_ levels in roots, and Nitrotetrazolium blue (NBT, 1 mg mL^−1^) (Sangon Biotech, Shanghai, China) was used for O_2_^−^ detection. DAB and NBT staining were performed as described by [32]. At least 30 root meristems for each staining were analyzed and imaged using an Olympus BX61 microscope (Olympus, Tokyo, Japan) equipped with differential interference contrast (DIC) optics, 20× UPlanSApo objective, and a CCD camera (Olympus DP74). For photo acquisition, Olympus cellSens software was used.

### 4.8. Image Analysis

Root length, meristem size, cell counts, DAB, and NBT images were investigated using ImageJ (http://www.imagej.nih.gov/ij/). DAB, and NBT stained images were quantified as described in [32]. Mean relative fluorescence for confocal images was calculated with ImageJ. In quantifying fluorescence, at least 15 seedlings per line were used.

### 4.9. Statistical Analysis

All experiments were performed at least three times. For statistical comparisons, we used Student’s *t*-test. Data shown are averages ± SD. Asterisks indicate significant differences compared with the control (*, *p* < 0.05, **, *p* < 0.01, ***, *p* < 0.001).

## 5. Conclusions

In this study, we investigated the effect of PAs on root development in *Arabidopsis* with the goal of identifying mechanisms by which PAs affect root growth. We explored changes in ROS accumulation and hormone signaling under conditions of ADC1/2 inhibition and Put depletion, and the results demonstrated that the effects of PAs on root phenotype are mediated by ROS and hormone signaling. Furthermore, the data generated by this study indicate that PA regulation of root phenotype is extremely complex, involving (1) intricate forward and reverse PA biosynthesis, (2) changes in ROS accumulation generated by PA catabolism and reverse biosynthesis and potentially by PA-mediated H_2_O_2_ scavenging, and (3) the complexities of hormonal crosstalk, resulting in a modified auxin distribution and CK response and changes to the ratio of auxin to CK signaling. This complexity is further evidenced by the contrasting changes in PA levels observed as ADC1/2 inhibition increased; by the non-linear trends in root growth, MZ size, and auxin response; and also by the differential regulation of PIN1 and PIN2 transporters.

Having identified candidate mechanisms linking PAs to root phenotype, future work requires more detailed investigations of these pathways under conditions of Put depletion. As noted earlier, although D-Arg treatment has been used in several studies to reduce Put, questions remain about the possible side-effects of D-Arg treatment. Therefore, the first step in future research will be to address this issue by developing ADC1/2 knockdowns and making comparisons with D-Arg treatment results for gene expression, hormone signaling, ROS accumulation, and phenotype. It would be ideal to be able to calibrate each knockdown with a specific D-Arg treatment level and, provided there is no significant difference between results for the knockdowns and D-Arg application, the two methods can be combined to deplete Put, utilizing the convenience of D-Arg that also allows fine-tuning of Put levels, which is difficult to achieve with knockdowns alone. The next step is to investigate mechanisms by which PAs modulate hormone signaling. The literature suggests links between PAs and auxin conjugation [37], CK biosynthesis [3,74], and CK degradation [37,38], and therefore the initial focus should be on components of these signaling pathways using genetic material. During this phase, possible ROS effects will be removed through the application of KI. A similar approach can be adopted for analyzing links between PAs and ROS.

Our initial results and the literature indicate that PA levels are closely inter-related and that it is difficult to predict how the perturbation of one PA will affect other PAs, hormone signaling, ROS accumulation, and phenotype. Therefore, another interesting research area would be a more detailed examination of PA balancing and downstream effects by treating WT and *adc1* and *adc2* single mutants and knockdowns with D-Arg and PAs, and observing them under stress conditions. Since ADC1/2 enzymes appear to have some level of redundancy given that single mutants do not exhibit phenotypes, the investigation of enzyme expression, localization, and redundancy under different conditions could also be carried out.

## Data Availability

Not applicable.

## Supplementary Materials

Supplementary Materials can be found at https://www.mdpi.com/article/10.3390/ijms22084094/s1.

## Abbreviations

PAs	Polyamines
ADC	Arginine decarboxylase
CK	Cytokinin
MZ	Meristem zone
Put	Putrescine
Spd	Spermidine
Spm	Spermine
Tspm	Thermospermine
uORFs	Upstream open reading frames
D-Arg	D-Arginine
CuAOs	Copper-containing amine oxidases
PAOs	Polyamine oxidase
RBOH	Respiratory burst oxidase homolog
ROS	Reactive oxygen species
CKX	Cytokinin oxidase
ARR	*Arabidopsis* response regulator
QC	Quiescent center
