# Supplementary material for: Putrescine Depletion Affects Arabidopsis Root Meristem Size by Modulating Auxin and Cytokinin Signaling and ROS Accumulation
Source: Int J Mol Sci. 2021 Apr 15;22(8):4094. doi: 10.3390/ijms22084094 (PMC8071467; doi:10.3390/ijms22084094)
Supplement: Supplementary file 1 [file ijms-22-04094-s001.zip › ijms-1153065-SI.pdf]

SUPPLEMENTARY INFORMATION

Table S1. Primers used in this study

**Primer name**	**Sequence**
CKX1-qF	GTTCCACATCCCTGGCTGAA
CKX1-qR	TGTTTCTTCCACTTGGATTGATTG
CKX4-qF	CATCTTTGTCCCGGGGTCTC
CKX4-qR	CGGTTGTTCCATTTGTTTCGG
CKX7-qF	GTTTGTCAACGGTGCTGACC
CKX7-qR	AAGAACCGACCCGCAAGATT
ARR5-qF	TCTACTCGCAGCTAAAACGC
ARR5-qR	AAGCCGAAAGAATCAGGACA
ACTIN2-qF	GACCAGCTCTTCCATCGAGAA
ACTIN2-qR	CAAACGAGGGCTGGAACAAG
